# Screening and Evaluation of Thiamethoxam Aptamer Based on Pressurized GO-SELEX and Its Sensor Application

**DOI:** 10.3390/bios13020155

**Published:** 2023-01-18

**Authors:** Yaqi Yue, De Zhang, Kangfei Tian, Dejiang Ni, Fei Guo, Zhi Yu, Pu Wang, Pei Liang

**Affiliations:** 1 Key Laboratory of Horticultural Plant Biology, Ministry of Education, College of Horticulture & Forestry Sciences, Huazhong Agricultural University, Wuhan 430070, China; 2 College of Optical and Electronic Technology, China Jiliang University, Hangzhou 310018, China

**Keywords:** GO-SELEX, ITC, colorimetric sensor, pesticide, aptamer

## Abstract

Thiamethoxam, a nicotinic pesticide used worldwide, can cause great harm to the environment and even to human health, and aptamers, known as chemical antibodies, have high affinity and specificity for the target, as well as great potential in detecting small molecules such as pesticides. In this paper, we report a highly sensitive biosensor system for thiamethoxam residue detection based on aptamer technology. After 15 rounds of screening with the pressurized GO-SELEX technology, we found that the aptamer libraries of the 5th and 9th rounds showed high affinity by the capture method. Four candidate aptamers were obtained by high-throughput sequencing and secondary structure prediction. Among them, the aptamer named Thi-5R-18 from the 5th round was demonstrated to possess the highest affinity by isothermal titration calorimetry, with a dissociation constant (Kd) of 4.935 × 10^−5^ M. The results of molecular docking showed that thiamethoxam and Thi-5R-18 were combined with bases G-15, A-19, and T-71 through hydrogen bonding and π–π interaction.Thi-5R-18 was used as a recognition element to construct a AuNPs colorimetric aptasensor, achieving an ultralow detection limit of 0.37 nM. More importantly, this colorimetric aptasensor can be used for quantitative detection of thiamethoxam on tea leaves, with a recovery of 96.94%~105.86%. This study provides a highly sensitive biosensor for detection of thiamethoxam residue.

## 1. Introduction

Thiamethoxam, the second generation of new nicotine toxic insecticide widely used all over the world [1,2], can destroy the normal conduction of insect central nerves and cause insect death [3] by binding with nicotinic acetylcholine receptor, leading to its popular application to control aphids, planthoppers, leafhoppers, whiteflies, and other pests. However, studies have shown that thiamethoxam has adverse effects on bees and fish in the ecosystem, such as impairing the flying ability of bees, reducing their return rate and survival rate [4,5], and damaging fish liver and affecting their reproductive capacity [6]. In the past 30 years, the use of thiamethoxam has caused great damage to the environment [7], as well as potential harm to mammals and even human beings [8]. Many countries and regions have issued laws and regulations to control pesticide use and set maximum residue limits (MRLs). Therefore, it is necessary to establish a rapid and accurate detection method for thiamethoxam. At present, the detection methods of thiamethoxam include high performance liquid chromatography (HPLC) [9], Liquid Chromatograph-Mass Spectrometer (LC-MS) [10,11], enzyme linked immunosorbent assay (ELISA) [12], etc. However, most of these methods require large and high-precision instruments, experienced operators, and time-consuming and cumbersome data processing, suggesting that it is imperative to develop a simple and convenient method for thiamethoxam detection.

The concept of nucleic acid aptamer was first proposed by Ellington and Tuerk in 1990 and since then, aptamers have been gradually and extensively developed [13,14]. Nucleic acid aptamers, known as chemical antibodies, are single stranded DNA or RNA. Aptamers that specifically bind to targets can be screened from synthetic libraries in vitro by Systematic Evolution of Ligands by Exponential Enrichment (SELEX) technology [15]. The targets of aptamers include proteins, cells, toxins, antibiotics, molecular markers, drugs, heavy metals, etc. [16]. Compared with antibodies, aptamers are easier to synthesize, more convenient to label, lower in immunogenicity, and higher in thermal stability [17]. There are mainly two types of SELEX technologies for pesticides: fixed and non-fixed SELEX, with fixed SELEX technology including microplate SELEX, magnetic bead SELEX, and capture SELEX, while non-fixed SELEX technology including GO-SELEX and capillary electrophoresis SELEX. Microwell plate SELEX couples pesticides with macromolecular proteins such as bovine serum albumin (BSA), or fixes small molecules to the microplate through a protein carrier, which can be more easily bound to ssDNA than pesticides. Magnetic bead SELEX connects the target molecules to the magnetic bead surface through a chemical coupling reaction and can quickly obtain the target binding sequence through magnetic separation. The magnetic bead (MB) surface can be modified with amino, carboxyl, streptavidin, etc. Targets with functional groups such as carboxyl, amino, and biotin can be coupled with MB, but there are few functional groups on the pesticide’s surface that can be coupled or modified. Therefore, the MB SELEX method to fix the target may change the target conformation and affect the binding site of ssDNA and the target. In practical applications, the target was in a free state, and the aptamers screened by MB SELEX may have a low affinity with the target. Capture SELEX fixes the library on the solid-phase carrier by using short strand ssDNA as the bridging sequence. In the presence of the target, the ssDNA binds to the target and allows its conformation to change, enabling ssDNA to detach from the solid carrier. Capture SELEX fixed libraries could effectively solve the impact of a fixed target. However, the fixation of the libraries will affect the diversity of oligonucleotide sequences, leading to unstable fixation efficiency, and thus the loss of sequences with high affinity and specificity to the target in the initial library. Capillary electrophoresis SELEX separates target-bound ssDNA from unbound ssDNA through mobility difference, which is efficient and highly selective, but is not applicable to small molecules with high mobility. Graphene oxide (GO) is obtained by a chemical method after strong oxidation of graphite [18]. The strong van der Waals force of GO makes it hydrophobic and easy to gather, and the oxygen-containing groups on the surface make it hydrophilic and dispersible, allowing GO to adsorb molecules containing aromatic rings through π-π force and hydrogen bonds [19]. The free ssDNA is separated from the target ssDNA complex through the hydrophobic interaction and unique adsorption on GO surface [20], and candidate sequences of aptamers are obtained by centrifugation. GO-SELEX can solve the problem that the targets do not have functional groups and have low molecular weight, making it suitable for screening of nucleic acid aptamers for small molecular targets.

Generally, GO-SELEX has the significant advantages of not requiring fixed targets or libraries, high screening efficiency, and simple operation. GO addition is a key factor affecting the adsorption of ssDNA, and during the screening process of GO-SELEX, the screening pressure usually increases with the increase of rounds, such as shortening the incubation time, increasing the proportion of GO/ssDNA or GO/target, etc. In this paper, we aimed to screen a thiamethoxam aptamer based on pressurized GO-SELEX and test the applicability of its sensor in thiamethoxam detection. Specifically, based on previous studies [21,22], aptamers of small pesticide molecular target of thiamethoxam were obtained by raising the additional amount of GO by an order of magnitude, and analyzed the impact of increased screening pressure on screening results. After 15 rounds of screening, an innovative capture method was set up to evaluate the affinity of the aptamer libraries of each round. High-throughput sequencing was carried out according to the rounds of affinity, and 4 candidate aptamers were found. The affinity of 4 aptamers was determined by isothermal titration calorimetry (ITC), and Thi-5R-18 was selected as the best aptamer. For thiamethoxam detection, the aptamer Thi-5R-18 was utilized as a component to identify targets and establish the AuNPs colorimetric sensor, which can be applied to thiamethoxam detection in actual tea leaves.

## 2. Materials and Methods

### 2.1. Materials

Thiamethoxam was obtained from MedChemExpress (Monmouth Junction, NJ, USA); GO from Shenzhen Suiheng Graphene Technology Co., Ltd. (Shenzhen, China); LA Taq from TaKaRa (Beijing, China); 50 bp DNA Ladder from Beijing Tsingke Biological Technology Co., Ltd. (Beijing, China); Agarose, 10 × TBE, 2 × Urea DNA Loading Buffer, DNA Urea-PAGE electrophoresis kit from Beijing Coolaber Technology Co., Ltd. (Beijing, China); UNIQ-10 Spin Column DNA Gel Extraction Kit for PAGE from Sangon Biotech (Shanghai, China) Co., Ltd. (Shanghai, China); and Tris-HCl, KCl, NaCl, and MgCl_2,_ 2-[(2-Hydroxy-1,1-bis(hydroxymethyl)ethyl)amino]ethanesulfonic acid (TES) (99% purity) from China Pharmaceutical Group Shanghai Chemical Reagent Co., Ltd. (Shanghai, China). All reagents were of analytical grade and ultrapure water was used throughout. Primers for screening aptamers and verifying effects, including FAM-labeled forward primers and Spacer18-labeled reverse primers (5′-ATAGGAGTCACGACGACCAGAA-3′; 5′-dA20-Spacer18-ATTAGTCAAGAGGTAGACGCACATA-3′), as well as unlabeled primers and library (5′-ATAGGAGTCACGACGACCAGAA-N_40_-TATGTGCGTCTACCTCTTGACTAAT-3′), were all obtained from Optimus Beijing Tsingke Biological Technology Co., Ltd. (Beijing, China) and purified by HPLC.

### 2.2. SELEX

ssDNA library (10μM) was dissolved in 1 × Binding buffer (50 mM Tris-HCl, 5 mM KCl, 100 mM NaCl, 1 mM MgCl_2_, pH 7.4), followed by boiling at 95 °C for 8 min and quenching immediately for 8 min. The adsorption capacity of GO to the target was verified as follows. Briefly, the GO/target mass ratios were set at 100:1, 200:1, 500:1, 1000:1, 2000:1, and 5000:1, respectively, followed by incubating GO and target together for 1 h and then centrifugation at 12000 rpm for 10 min. The supernatant was collected three times to measure the light absorbance value. Next, the GO/ssDNA mass ratio was optimized by setting, the GO/ssDNA mass ratios at 100:1, 200:1, 300:1, 400:1, 500:1, 1000:1, 2000:1, 3000:1, 4000:1, and 5000:1, respectively, followed by incubating GO and ssDNA together for 1 h, centrifugation at 12000 rpm for 10 min, and collecting the supernatant three times to measure the concentration of ssDNA. In the first round of screening, 5000:1 and 500:1 were selected as the mass ratios of GO and ssDNA. Meanwhile, the library was mixed with thiamethoxam solution and incubated at room temperature for 1 h, followed by adding 15 mg/mL GO, incubation for 1 h, and centrifugation at 12000 rpm for 10 min to collect the supernatant as the PCR template. The 50 μL PCR system mixture included 2.5 μL forward primer (10μM), 2.5 μL reverse primer (10 μM), 0.5 μL Takara La Taq, 5 μL La Taq buffer, 8 μL dNTP Mix and 31.5 μL PCR template. The PCR was performed under the conditions of 94 °C pre-denaturation for 5 min, 30 cycles of 98 °C denaturation for 45 s, 61 °C annealing for 15 s, 72 °C extension for 15 s, and 72 °C final extension for 1 min, performed successively. The same amount of 2 × urea loading buffer was added into the PCR product and was boiled at 95 °C for 3 min. After purification and recovery by 8% Urea-PAGE electrophoresis, the FAM-labeled ssDNA was used as the aptamer library for the next round of aptamer screening. A total of 15 rounds of aptamer screening were conducted.

### 2.3. Round Affinity Analysis

For round affinity analysis, each round of the aptamer library was used as a template, and biotin-labeled forward primer and dA20-Spacer18-labeled reverse primer were used for PCR. PCR products were purified and recovered by 8% Urea-PAGE to obtain a biotin-labeled aptamer library. After denaturation and renaturation, the aptamer library was attached to streptavidin beads, followed by adding an equal concentration of thiamethoxam to the complex solution and incubated for 1 h. Next, the supernatant was separated by magnetic absorption, followed by cleaning the magnetic beads twice with TES, adding ultrapure water in a 60 °C metal bath for 5 min, and then measuring absorption value of the supernatant at 255 nm.

### 2.4. HTS and Secondary Structure Prediction

The aptamers obtained in the 5th and 9th rounds were tested by PCR using unlabeled common primers. The purified product was sent to Novogene Co. Ltd. (Beijing, China), and the Illumina Platform was used for library construction and HTS. The results of HTS were analyzed by self-compiled programs. The secondary structure and free energy of the aptamers were predicted through online websites (www.unafold.org, accessed on 11 October 2022).

### 2.5. Measurement Dissociation Constant (Kd) by ITC

The Kd of aptamer sequence was measured by ITC. Briefly, the sample was degassed for 10 min at a vacuum degree above 400 mmHg. Both the aptamers and pesticide were dissolved in 1 × binding buffer, followed by adding 350 μL of 25 μM aptamers to the titrator and 50 μL of 1mM thiamethoxam to the sample cell. ITC was performed under the following conditions: total titration number, 20; volume of each titration, 2.5 μL; time, 200 s; reaction temperature, 25 °C; and stirring speed, 250 rpm. After running, the titration results were analyzed by Launch NanoAnalyze software to obtain the Kd of each aptamer.

### 2.6. Analysis and Verification of the Binding of Aptamer and Thiamethoxam

The secondary structure file of the aptamer was saved in Vienna output format, which was used to build the 3D structure of the aptamer (http://biophy.hust.edu.cn/new/, accessed on 6 January 2023). The 3D structure file of thiamethoxam molecule was download from the website (https://pubchem.ncbi.nlm.nih.gov/, accessed on 6 January 2023). AutoDock 4.2.6 software was used to predict the docking of aptamer and thiamethoxam molecule, and OpenBabel 2.4.1 software was used to convert the docking result from pdb format to pdbqt format. The possible interaction sites between the aptamer and thiamethoxam molecules were analyzed, and PyMoL 1.7.6 software was used to visualize the molecular docking results. After the docking sites of thiamethoxam and the aptamer were known, bases were truncated at both ends of the sequence (Figure S1A). The Kd of truncated sequence was verified by ITC.

### 2.7. Thiamethoxam Detection with AuNPs Colorimetric Sensor

The AuNPs colorimetric sensor was constructed with Thi-5R-18 to verify the practicability of the aptamer. The AuNPs were prepared as reported by Zheng et al. [23]. Briefly, 10 μM aptamers (5 μL) was added to each EP tube and incubated with 250 μL of different concentrations of thiamethoxam for 10 min, followed by adding 135 μL of nano particles and incubating for 5 min. After adding 70 μL of 0.25 M NaCl rapidly to the solution and incubating for 5 min, 200 μL of the obtained solution was collected and placed in an enzyme standard plate for 200 nm~800 nm spectral scanning. The linear regression equation was established between the concentration of thiamethoxam and the absorbance value of the mixed system. The specificity of the aptamer was tested with 10^−8^ M of thiamethoxam and 10^−7^ M of dinotefuran, admire, clothianidin, and glyphosate. Meanwhile, the AuNPs colorimetric sensor was used to detect thiamethoxam in real samples by spraying 300 μL of different concentrations of thiamethoxam (20nM, 40nM, 80nM, and 100nM) on tea leaves, followed by drying at room temperature, washing with 300 μL ultrapure water to obtain thiamethoxam solution, and then using the aptasensor to obtain the thiamethoxam concentration by measuring the A_650/520_.

## 3. Results and Discussion

### 3.1. Screening of Thiamethoxam Aptamers

**Figure 1:**
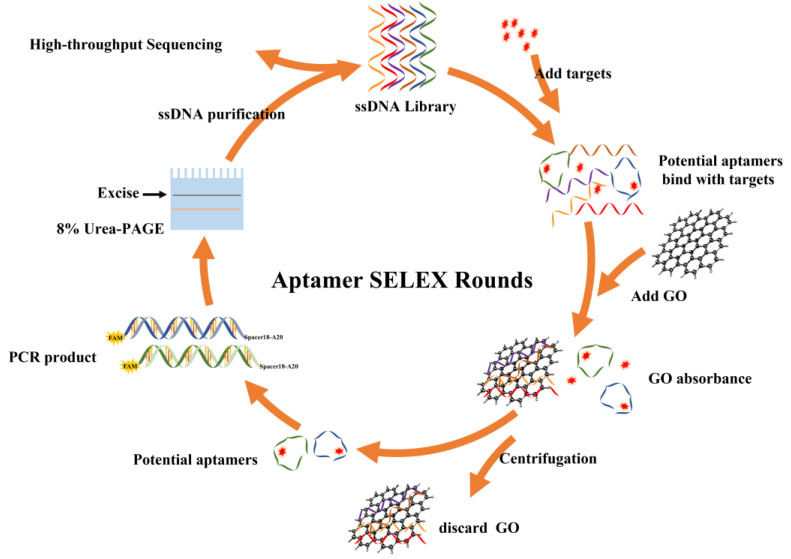
Schematic diagram for screening thiamethoxam aptamers by GO-SELEX.

In this study, GO-SELEX technology was used to screen thiamethoxam aptamers. During screening, the ssDNA library was first incubated with thiamethoxam to facilitate the combination of thiamethoxam and ssDNA to form a complex. After adding GO, a large amount of ssDNA with no affinity to thiamethoxam was captured on the GO surface through π-π stacking, and only a small amount of target-bound ssDNA remained in the supernatant. After PCR amplification, product recovery, and purification, ssDNA was obtained as a secondary library to enter the next round of screening (Figure 1).

**Figure 2:**
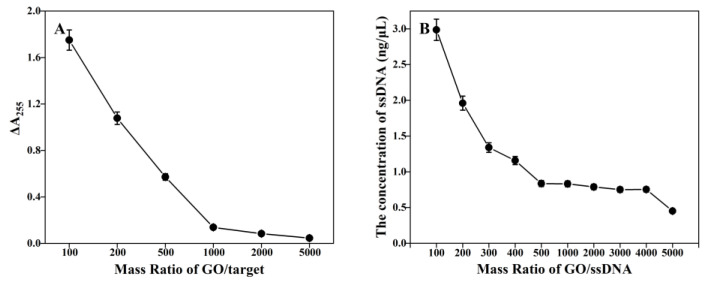
(**A**) Optimization of the mass ratio of GO and target. ΔA255 indicated the difference value between the absorbance value at 255nm of the supernatant (GO/target) and the control (GO/1× Binding buffer). (**B**) Optimization of the mass ratio of GO and ssDNA. The concentration of ssDNA indicated the content of free ssDNA in the supernatant.

GO-SELEX was suitable for screening the aptamers of targets which cannot be absorbed by GO. ΔA255 was the difference value between the absorbance value at 255 nm of the supernatant incubated with GO/target and GO/1 × Binding buffer (control). As shown in Figure 2A, after adding GO and the target in the incubation system at the ratio of 5000:1, free target still existed in the solution. Based on the previous report [21] and cost, 500:1 was used as the mass ratio of GO/target. 

The key to aptamer screening is to obtain and isolate high affinity binding sequences from random ssDNA libraries. Since different lengths of ssDNA show different binding kinetics on the GO surface [24], the GO/ssDNA mass ratio was optimized before GO-SELEX experiments, enabling GO to fully adsorb ssDNA. Commonly, the GO/ssDNA quality ratio was between 500:1 and 1000:1 [21,25], allowing the ssDNA concentration to reach the baseline level. In Figure 2B, the nucleic acid concentration was seen to decrease with the increase of GO/ssDNA, and at the mass ratio of 500:1, the DNA content in the solution reached the first equilibrium point, indicating that this concentration reached the baseline level, consistent with previous studies [21]. At GO/ssDNA mass ratio was between 500:1~4000:1, although the nucleic acid concentration could reach the baseline level, there was still free ssDNA not bound to the target, which would become non-specific enrichment sequences and existed in each round of the library, thus affecting the screening efficiency. When the mass ratio further increased to 5000:1, the concentration of ssDNA decreased significantly and the content of free ssDNA in the solution decreased to the minimum at the same time. This experiment aimed to compare the difference between pressurized SELEX and ordinary SELEX, so the high pressure GO/ssDNA ratio was selected for the SELEX process. In the subsequent SELEX process, 5000:1 was used as the mass ratio of GO/ssDNA. In this study, a FAM-labeled forward primer and a dA20-spacer18-labeled reverse primer were used to separate the aptamer sequence by 8% Urea-PAGE electrophoresis, with FAM-labeled forward primer PCR product being used in the next round of the aptamer library. In this experiment, 15 rounds of screening were conducted to explore the effect of pressurized GO-SELEX on the round affinity.

### 3.2. Round Affinity Analysis

**Figure 3:**
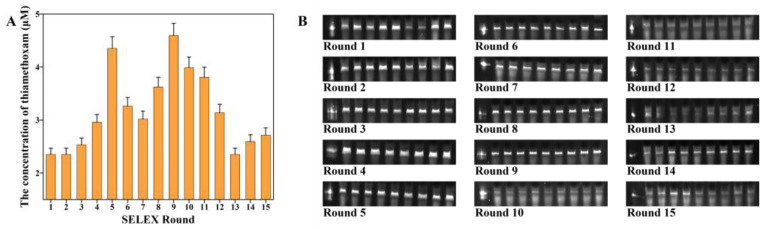
(**A**) The concentration of eluted thiamethoxam in each round. (**B**) Result for each round of 8% urea-PAGE. The corresponding electrophoretic bands from left to right are self-made 87nt marker, FAM-labeled 1~15 round candidate aptamer bands.

The round affinity between aptamer library and target is usually determined by the recovery rate [26], which was expressed by the ratio of ssDNA concentration before incubation and after incubation. Thiamethoxam has a maximum absorption wavelength at 255 nm, close to the maximum absorption wavelength of ssDNA. As the measured ssDNA concentration was inaccurate, this study used an innovative capture method to measure the affinity of each round. Specifically, each round of biotin-labeled aptamer library was fixed onto the magnetic beads with streptavidin magnetic beads, allowing the aptamers to capture the free targets in the solution. The more affinity sequences in a round, the more targets could be captured, and the higher the absorbance at 255 nm. As shown in Figure 3A, the proportion of ssDNA with affinity to thiamethoxam in the library was low at initial screening, and after several rounds of screening, the affinity of ssDNA was gradually enriched. The aptamers obtained from the 5th and 9th rounds had the highest affinity. With the further increase of rounds, the affinity showed no further improvement, and in Figure 3B, the result of 8% Urea-PAGE gel also showed that the brightness of gel bands was unstable from the 10th round. The brightness of gel bands indirectly indicated the DNA content. The quality ratio of GO/ssDNA in the screening process was 5000:1, which was one order of magnitude higher than that in general GO-SELEX experiments, hence the higher screening pressure inferring that the adsorption of GO on ssDNA surface was unstable, which would lead to the loss of affinity sequences.

### 3.3. HTS and Secondary Structure Analysis of Sequences

**Figure 4:**
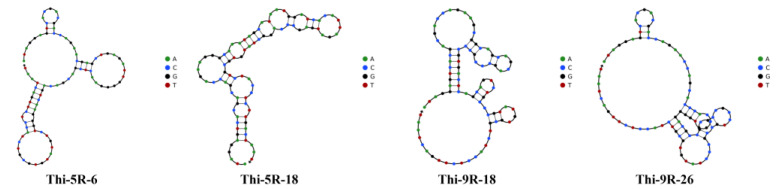
The secondary structures of aptamer candidates.

The number of aptamer sequences screened in this study was more than one million and the first 30 sequences with the highest enrichment degree were selected as candidate aptamers in each round, which were named Thi-5R-1~Thi-5R-30 and Thi-9R-1~Thi-9R-30, according to the enrichment degree. Candidate aptamers were selected based on the representative secondary structure (Figure 4) and low free energy sequences (Table 1). Low free energy represented the stability of the secondary structure formed by the sequences. Each candidate sequence of aptamer contained the stem ring structure formed by hydrogen bond connection within the molecule, which was the structural basis for combination with thiamethoxam. 

### 3.4. Measurement of Sequence Affinity by ITC

**Figure 5:**
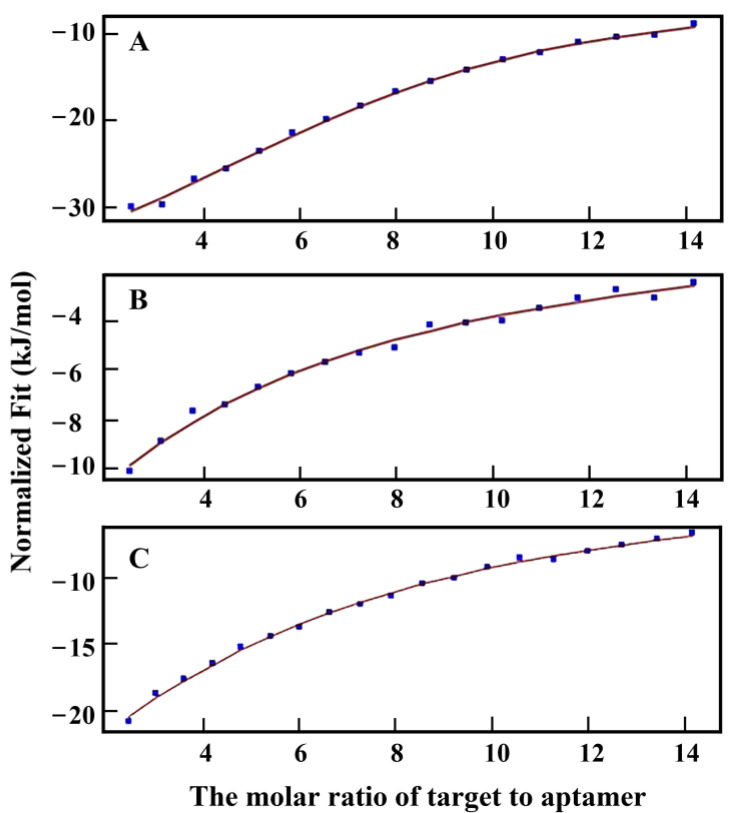
ITC analysis of (**A**) Thi-5R-18 (**B**) FAM-Thi13 (**C**) seq.20 with thiamethoxam.

In this study, the affinity of candidate aptamers was evaluated by Kd values calculated through the ITC. ITC could directly measure the heat released in the process of biomolecular binding, and the experimental data were expressed in the form of thermogram, which could accurately obtain the complete thermodynamic information of biomolecular interaction. The potential molecular interaction mechanism could be clarified by analyzing the ITC data. According to the ITC results (Figure 5), the Kd value was 4.935 × 10^−5^ M for Thi-5R-18 combined with the target to release heat, and the Kd values of two published thiamethoxam aptamers FAM-Thi13 [27] and seq.20 [28] were 3.661 × 10^−4^ M and 2.115 × 10^−4^ M, respectively, indicating that the aptamer Thi-5R-18 in this paper had the lowest Kd value and the highest affinity among the three aptamers. Therefore, Thi-5R-18 was used as the aptamer for thiamethoxam detection in the subsequent sensor performance test.

### 3.5. The Binding of Aptamer and Thiamethoxam

The binding model of Thi-5R-18 and thiamethoxam was further analyzed by molecular docking. The results of molecular docking (Figure S1B) showed that the small molecule of thiamethoxam and Thi-5R-18 were combined with hydrogen bond and π–π interaction. Thiamethoxam was combined with bases G-15, A-19, and T-71. Further analysis showed that the oxygen atom at the end of G-15 of Thi-5R-18 formed a hydrogen bond with the 1^st^ oxygen atom of thiamethoxam (The bond length was 2.8 Å). The oxygen atom at the end of T-71 of Thi-5R-18 formed a hydrogen bond with the 7^th^ oxygen atom of thiamethoxam (The bond length was 3.7 Å). The benzene ring of thiamethoxam formed π–π bond with the benzene ring of T-71 (The bond length was 4.7 Å). These hydrogen bonds and π–π bond further stabilized the binding of thiamethoxam to Thi-5R-18. Previous studies have shown that binding may also be related to van der Waals force and electrostatic force [27]. A total of 19 bases that included G-15 and A-19 and 17 bases that included T-71 at both ends of the sequence were cut off. Figure S2 showed that the Kd value of truncated aptamer sequence measured by ITC could not be fitted. It was confirmed that after deletion of the binding site, the affinity of the aptamer disappeared.

### 3.6. Detection of Thiamethoxam by Aunps Colorimetric Sensor

**Figure 6:**
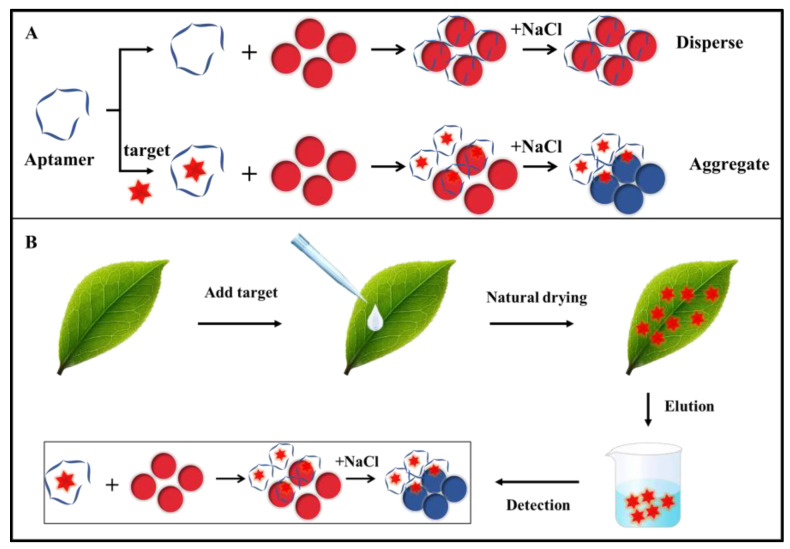
Schematic diagram of (**A**) color development principle of AuNPs and (**B**) surface detection of actual tea leaf samples.

**Figure 7:**
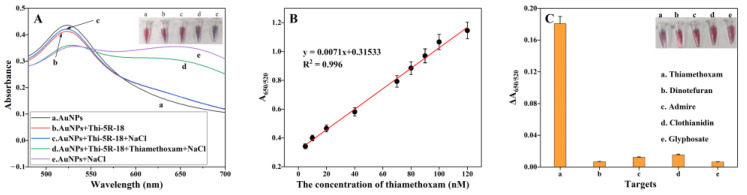
(**A**) UV-vis spectroscopy of AuNPs under different conditions. (**B**)The linear relationship between the value of A_650/520_ and the concentration of thiamethoxam. (**C**) The value of A_650/520_ for different targets.

To verify the applicability of the aptamer Thi-5R-18 in thiamethoxam detection, a colorimetric sensor based on AuNPs was constructed with Thi-5R-18 as the recognition element. As displayed in Figure S3, the size of nanoparticles was relatively uniform with an average particle size of 15.90 nm. As previously reported, there was a large amount of positive charge on the surface of AuNPs [29], and when NaCl was added, the Cl^−^ could destroy the charge balance on the surface of AuNPs, making the nanoparticles aggregate and the solution blue. However, the aptamers adsorbed on the surface of AuNPs through electrostatic adsorption would keep the nanoparticles in a dispersed state, enabling the solution to stay red and the nanoparticles not to aggregate even after adding NaCl (Figure 6A). After adding the target, the targets were combined with aptamers and left on the surface of the AuNPs, allowing the nanoparticles to aggregate and the solution to turn blue after adding NaCl. As shown in Figure 7A, 520 nm was the maximum absorption wavelength of AuNPs, and the aggregation of particles reduced the light absorption value here. After adding the target, the absorbance value declined at 520 nm but increased at 650 nm. In Figure 7B, a good linear correlation was shown between the intensity ratio of light absorption values at 650 nm and 520 nm (A_650/520_) and the concentrations of thiamethoxam. The linear response range of the colorimetric sensor was 5 to 120nM. The linear regression equation was calculated as y = 0.0071x + 0.31533 and the limit of detection (LOD) of the sensor was 0.37 nM (with calculation details shown in supporting information), indicating the high sensitivity of the aptsensor system. Figure 7C showed the specificity test result of the thiamethoxam aptamer based on the colorimetry method. Four pesticides with structures similar to thiamethoxam were detected by the aptsensor system and the binding ability of the aptamer to the four targets was less than one tenth of that of thiamethoxam, although their concentration was ten times higher than that of thiamethoxam, indicating the high specificity of the aptasensor system for thiamethoxam.

In order to test the applicability of this aptamer colorimetric sensor in actual samples, the thiamethoxam pesticide was sprayed on the leaf surface and eluted for colorimetric detection after natural drying. The concentration of thiamethoxam after elution was 20, 40, 80, and 100 nM (Figure 6B), respectively. The results (Table 2) showed that the recovery was 96.94%~105.86% and RSD (Relative Standard Deviation) was 0.41%~3.76%, implying that the constructed aptamer colorimetric sensor had good repeatability and reliability.

## 4. Conclusions

In this study, non-immobilized GO-SELEX was used to screen ssDNA aptamers that specifically bind to thiamethoxam. In the screening process, the screening pressure was improved by increasing the additional amount of GO. The 5th and 9th rounds of libraries showed the highest affinity using a capture method. This method was innovatively used to determine the affinity of round libraries, and effectively solved the problem that the maximum absorption wavelength was close between target and ssDNA. Aptamer Thi-5R-18 obtained from the 5th round can specifically bind to the target and its affinity was proved to be the best by ITC, demonstrating that high screening pressure was conducive to shortening screening rounds and reducing experimental cost. The results of molecular docking showed that thiamethoxam and Thi-5R-18 were combined with bases G-15, A-19, and T-71 through hydrogen bonding and π–π interaction, which was also verified by ITC. Moreover, a colorimetric aptasensor based on AuNPs was constructed by the aptamer Thi-5R-18, with the linear regression equation y = 0.0071x + 0.31533 and an LOD of 0.37 nM. Furthermore, the aptasensor system was applied to actual sample detection, achieving the recovery of 96.94%~105.86% and the RSD of 0.41%~3.76%, suggesting that the aptasensor could be used for rapid and sensitive detection of thiamethoxam residues.

## Figures and Tables

**Table 1:** Sequences of aptamer candidates.

Aptamer	40nt Sequences (5′-3′)	Free Energy (kcal/mol)
Thi-5R-6	CGAGCTGAGATTGGGGAACTCGACGACAGTCAAGGGTCTG	−9.78
Thi-5R-18	GGGCAAATAGCATAATGGATCACATTAGATGAGCCCAGGC	−7.4
Thi-9R-18	GCGGCAGCAGCAGCCCGCCCGTGACTCAGCAGTCTGCCCG	−11.4
Thi-9R-26	CCAGCACCCACCGGTGGGGACGCCGCGCCGCCTGCCGCCC	−8.24

**Table 2:** Determination of thiamethoxam pesticide in spiked real samples.

Sample	Spiked Concentration (nM)	Measured Concentration ^a^ (nM)	Recovery (%)	RSD (%)
1	100	105.40	105.40	1.88
2	80	84.69	105.86	0.41
3	40	38.78	96.94	3.76
4	20	19.61	98.03	0.94

^a^ Mean values were calculated from three determinations.

## Data Availability

Not applicable.

## Supplementary Materials

The following supporting information can be downloaded at: https://www.mdpi.com/article/10.3390/bios13020155/s1, Figure S1: (A) Secondary structures of Thi-5R-18. The base cutting sites were labeled by scissors. G-15, A-19, T-71 were the binding sites. (B) Molecular docking of thiamethoxam and Thi-5R-18. Figure S2: ITC analysis of truncated Thi-5R-18 with thiamethoxam. Figure S3: (A) TEM images of AuNPs. (B) Size distribution of AuNPs.
