# Supplementary material for: Screening and Evaluation of Thiamethoxam Aptamer Based on Pressurized GO-SELEX and Its Sensor Application
Source: Biosensors (Basel). 2023 Jan 18;13(2):155. doi: 10.3390/bios13020155 (PMC9953353; doi:10.3390/bios13020155)
Supplement: Supplementary file 1 [file biosensors-13-00155-s001.zip › biosensors-2062679-supplementary.pdf]

Calculation of the limit of detection (LOD)

The limit of detection (LOD) is the point at which the measured value is greater than the uncertainty associated with it. In this study, the LOD is calculated according to the provisions of the International Union of Pure and Applied Chemistry (IUPAC):

$$X_L = X_B + k \times S_B \tag{S1}$$

In this equation, X_L represents the minimum value of $A_{650/520}$ that can be detected, X_B represents the average value of $A_{650/520}$ for the blank sample, S_B represents the standard deviation, and k is a numerical factor chosen according to the desired confidence level. According to the linear regression equation *y=0.0071x+0.31533* (R^2=99.6%), the LOD could be calculated as 0.37 nM.

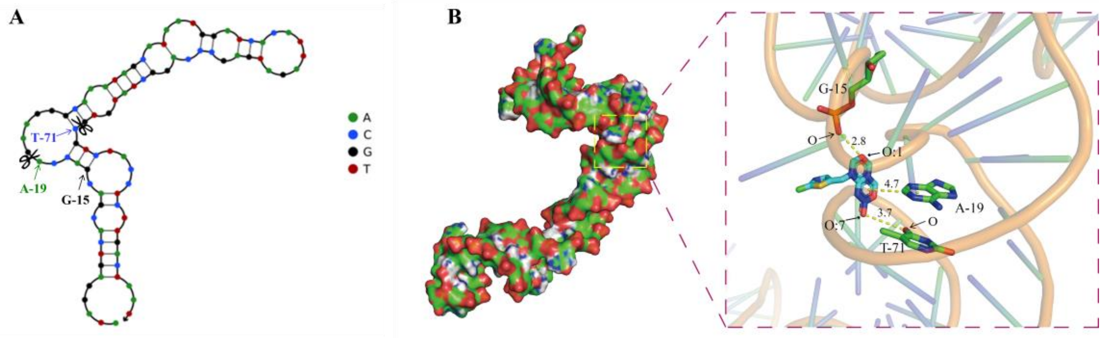

Figure S1. (**A**) Secondary structures of Thi-5R-18. The base cutting sites were labeled by scissors. G-15, A-19, T-71 were the binding sites. (**B**) Molecular docking of thiamethoxam and Thi-5R-18.

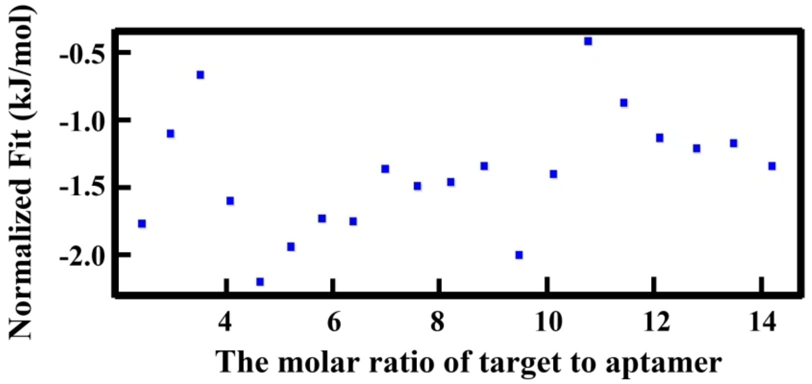

Figure S2. ITC analysis of truncated Thi-5R-18 with thiamethoxam.

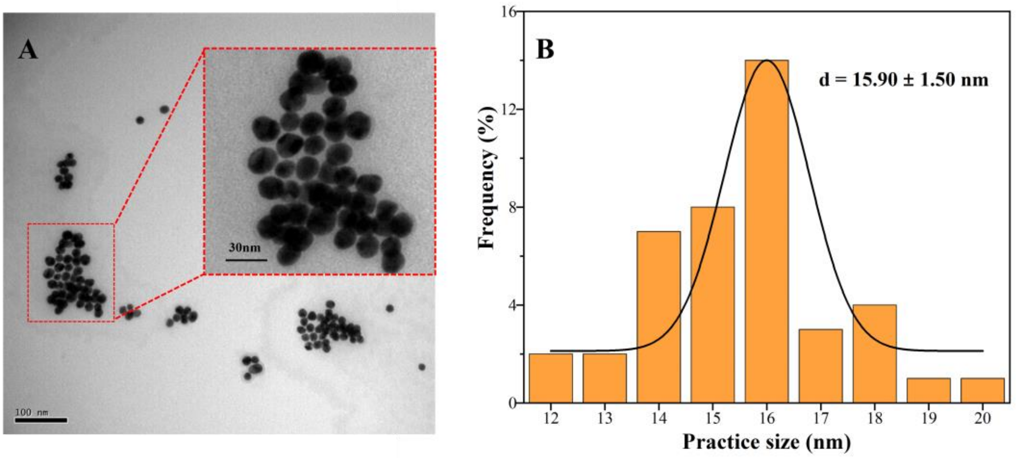

Figure S3. (**A**) TEM images of AuNPs. (**B**) Size distribution of AuNPs.
